# New Shear Horizontal (SH) Surface-Plasmon-Polariton-like Elastic Surface Waves for Sensing Applications

**DOI:** 10.3390/s23249879

**Published:** 2023-12-17

**Authors:** Piotr Kiełczyński

**Affiliations:** Institute of Fundamental Technological Research, Polish Academy of Sciences, ul. Pawińskiego 5B, 02-106 Warsaw, Poland; pkielczy@ippt.pan.pl

**Keywords:** ultrasonic sensors, metamaterial elastic waveguides, negative elastic compliance, shear horizontal (SH) elastic surface waves, SPP electromagnetic waves, phase and group velocity, complex power flow, penetration depth, elastic-electromagnetic analogies

## Abstract

The advent of elastic metamaterials at the beginning of the 21st century opened new venues and possibilities for the existence of new types of elastic (ultrasonic) surface waves, which were deemed previously impossible. In fact, it is not difficult to prove that shear horizontal (SH) elastic surface waves cannot exist on the elastic half-space or at the interface between two conventional elastic half-spaces. However, in this paper we will show that SH elastic surface waves can propagate at the interface between two elastic half-spaces, providing that one of them is a metamaterial with a negative elastic compliance $s_{44}\left(\omega \right)$. If in addition, $s_{44}\left(\omega \right)$ changes with frequency ω as the dielectric function ε(ω) in Drude’s model of metals, then the proposed SH elastic surface waves can be considered as an elastic analogue of surface plasmon polariton (SPP) electromagnetic waves, propagating at a metal-dielectric interface. Due to inherent similarities between the proposed SH elastic surface waves and SPP electromagnetic waves, the new results developed in this paper can be readily transferred into the SPP domain and vice versa. The proposed new SH elastic surface waves are characterized by a strong subwavelength confinement of energy in the vicinity of the guiding interface; therefore, they can potentially be used in subwavelength ultrasonic imaging, superlensing, and/or acoustic (ultrasonic) sensors with extremely high mass sensitivity.

## 1. Introduction 

Elastic surface waves that exist in solid waveguides seemingly have very little in common with surface plasmon polariton (SPP) electromagnetic waves propagating in metal-dielectric waveguides. However, with the advent of new elastic metamaterials, this assertion must be revisited. 

Indeed, one can argue that the invention of metamaterials was one of the most significant events in physics at the turn of the XX and XXI centuries [1,2]. In fact, metamaterials challenged many tacit assumptions and beliefs accumulated over decades about the properties of matter and wave motion herein. Combining basic research with a judicious engineering design, researchers devised many new materials with unprecedented properties. In the domain of elastic media, we observed the emergence of elastic metamaterials with a negative mass density [3,4,5], anisotropic mass density [6], negative elastic constants [7,8], etc. Not surprisingly, these new properties opened possibilities for the existence of new types of acoustic waves, which were previously considered impossible.

To date, it has been commonly agreed that shear horizontal (SH) elastic surface waves cannot exist at the interface between two elastic half-spaces [9]. In this study we challenge the above assertion, showing that SH acoustic (ultrasonic) surface waves can efficiently propagate at the interface between two elastic-half-spaces, providing that one of them is elastic metamaterial with special properties, i.e., with a negative shear elastic compliance.

Inspired by the newly developed elastic metamaterials, we propose in this paper a new type of shear horizontal (SH) elastic surface waves that were impossible in conventional elastic waveguides [9]. The new SH elastic surface waves can propagate at the interface between two elastic half-spaces one of which is a metamaterial with a negative elastic compliance $s_{44}\left(\omega \right)<0$. If, in addition, the compliance $s_{44}\left(\omega \right)$ changes with angular frequency ω as the dielectric function ε(ω) in Drude’s model of metals, the proposed SH elastic surface waves can be considered as direct elastic analogues of Surface Plasmon Polariton (SPP) electromagnetic waves propagating at a metal-dielectric interface.

As a result, special attention was paid in this paper to similarities between the newly proposed SH elastic surface waves and the electromagnetic surface waves of the surface plasmon polariton (SPP) type, propagating at a dielectric-metal interface [10,11,12]. In fact, SPP surface waves are transverse magnetic (TM) electromagnetic modes with only one transverse component, namely the magnetic field $H_{3}$ that is analogue of the SH particle velocity $v_{3}$ of the new proposed SH elastic surface wave. It is noteworthy that both types of waves share one crucial property, i.e., very strong subwavelength decay in the transverse direction away from the guiding interface $x_{2}=0$, especially in the metal and elastic metamaterial half-spaces.

Due to strong formal similarities between the SPP electromagnetic surface waves and the new proposed SH elastic surface waves, most of the results obtained in this paper can be transferred verbatim into the SPP domain by mutual substitution of the appropriate symbols. However, a transition from the SPP domain into the SH elastic surface wave domain can be very beneficiary for the latter due to a very large number of interesting new phenomena observed already in the SPP domain, such as trapping of light (zero group velocity) [13], transformational optics systems [14] or nonreciprocal and topological waveguides [15], just to name a few. Therefore, the proposed new SH elastic surface waves may open new fascinating possibilities to control wave phenomena occurring in elastic solids.

The new SH elastic waves have the character of surface waves since they decay exponentially in the direction of axis $x_{2}$, perpendicular to the interface ($x_{2}=0$) and perpendicular simultaneously to the direction of propagation $x_{1}$.

Another advantage of the proposed new SH elastic surface waves is the fact that they have only one component of the mechanical displacement $u_{3}$ (along axis $x_{3}$), which is completely uncoupled with the remaining components of mechanical vibrations, such as longitudinal (L, along axis $x_{1}$) and shear vertical (SV, along axis $x_{2}$). Multimodal coupling may be a significant problem in conventional bulk ultrasonic devices [16,17].

The proposed new SH elastic surface waves can have deep subwavelength penetration depth, in both half-spaces of the waveguide, therefore they offer a potential for applications in subwavelength acoustic imaging, superlensing, and/or acoustic sensors with extremely large sensitivity, analogously to their SPP counterparts in electromagnetism. These are very attractive properties of the newly discovered SH elastic surface waves.

The frequency range, in which the new SH elastic surface wave can propagate, covers practically the range from several kHz to several MHz. The maximum wave frequency $\omega_{sp}/2\pi$ depends on the resonant frequency of local resonators $\omega_{p}$ and is given by Formula (24) in Section 3.3. For example, when an exemplary waveguide structure depicted in Section 2.1 consists of (1) the metamaterial half-space ($x_{2}\leq 0$) composed of ST-Quartz with embedded local resonators with a selected resonant frequency $\omega_{p}/2\pi =1\;\mathrm{MHz}$ and (2) a conventional PMMA elastic half-space ($x_{2}\geq 0$), the maximum frequency of the new SH elastic surface waves equals approximately $\omega_{sp}/2\pi =143\;\mathrm{kHz}$, according to the Formula (24) in Section 3.3.

The proposed new SH elastic surface waves have a potential for very high resolution (of the order of micrometers) using relatively low ultrasonic frequencies (of the order of a few MHz). So far, using the conventional ultrasonic waves and imaging systems a comparable resolution could be achieved using frequencies of the order of 1 GHz. Needless to say, such a frequency range is still quite difficult to handle in ultrasonic practice.

The concentration of the elastic energy near the guiding interface can be of crucial importance in subwavelength acoustic imaging, acoustic energy harvesting as well as in miniaturized modern ultrasonic devices at the micro and nano-scale.

Several analytical equations developed in this paper are new and have not yet been published elsewhere. As a result, we hope that they can provide fresh physical insight into the wave phenomena occurring in both domains, namely SPP electromagnetic waves and SH elastic surface waves, proposed in this paper. For example, Equations (30), (33), (36) and (37) that relate complex power flow with penetration depths in both half-spaces of the waveguide, were to the best of our knowledge not yet published in the literature.

Due to their close similarity with the electromagnetic SPP waves the proposed new ultrasonic waves are characterized by a large confinement of acoustic energy near the surface. For this reason, these newly discovered SH acoustic waves can constitute the basis of a new generation of acoustic (ultrasonic) sensors with a giant mass sensitivity.

The layout of this paper is as follows. Section 2.1 introduces the geometry and material parameters of two half-spaces forming the metamaterial waveguide. Section 2.2 presents the metamaterial half-space with a negative elastic compliance $s_{44}^{\left(1\right)}\left(\omega \right)<0$. In Section 2.3 we derive a complete quantitative model of a metamaterial, whose elastic compliance $s_{44}\left(\omega \right)$ obeys the Drude relation. How to fabricate the elastic metamaterial with Drude-like elastic compliance is discussed in Section 2.4. Mechanical displacement $u_{3}$ and shear stresses $\tau_{13},\tau_{23}$ are subject to Section 3.1. Boundary conditions and the dispersion equation of the new SH elastic surface waves are presented in Section 3.2. The analytical formula for the wavenumber k(ω) was derived in Section 3.3. The formulas for the phase $v_{p}\left(\omega \right)$ and group $v_{g}\left(\omega \right)$ velocities were developed, in Section 3.4 and Section 3.5, respectively. The equations for the penetration depth in both half-spaces of the waveguide are given in Section 3.6. The net active power flow $P_{1}\left(\omega \right)$, in the direction of propagation $x_{1}$, was determined in Section 3.7. The average reactive power flow $P_{2}\left(\omega \right)$, in the transverse direction $x_{2}$ was analyzed in Section 3.8. The correspondence between SPP electromagnetic surface waves and the proposed new SH elastic surface waves is outlined in Section 4. The results of numerical calculations and the corresponding figures are presented in Section 5. The discussion and conclusions are the subject of Section 6 and Section 7, respectively.

## 2. Physical Model

### 2.1. Geometry and Material Parameters of the Waveguide

**Figure 1:**
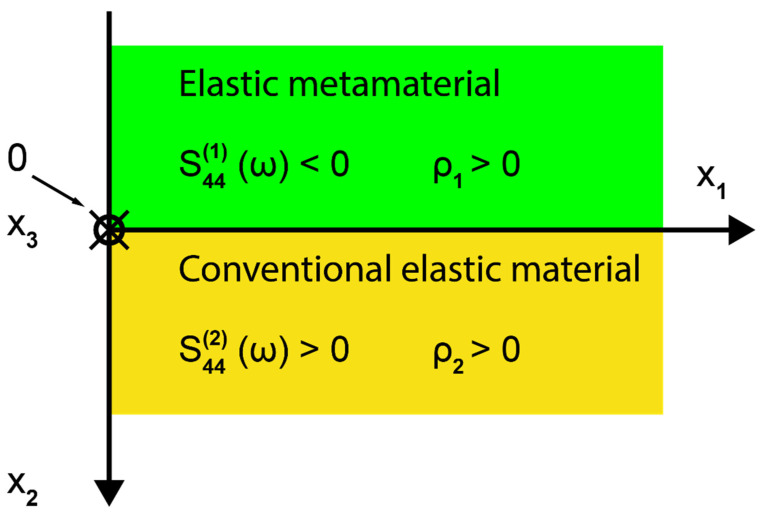
Cross-section of the waveguide supporting the new proposed SH elastic surface waves, propagating in the direction $x_{1}$, with exponentially decaying fields in the transverse direction $x_{2}$. The conventional elastic half-space ($x_{2}\geq 0$) is rigidly bonded to the metamaterial elastic half-space ($x_{2}<0$) at the interface $x_{2}=0$. The mechanical displacement $u_{3}$ of the new SH elastic surface waves is polarized along $x_{3}$ axis.

The geometry of the waveguide supporting new SH elastic surface waves is sketched in Figure 1. The waveguide consists of two semi-infinite elastic half-spaces, one of which is a conventional elastic material $\left(x_{2}\geq 0\right)$ and the second an elastic metamaterial $\left(x_{2}<0\right)$ with a negative elastic compliance $s_{44}^{\left(1\right)}\left(\omega \right)<0$, which is a function of angular frequency ω. By contrast, the densities $\left(\rho_{1},\rho_{2}\right)>0$ in both half-spaces as well as the elastic compliance $s_{44}^{\left(2\right)}>0$ in the conventional elastic material are positive and frequency independent (see Figure 1).

Two elastic half-spaces, rigidly bonded at the interface $x_{2}=0$, are uniform in the direction $x_{3}$, therefore all field variables of the new SH elastic surface wave will vary only along the transverse direction $x_{2}$, i.e., as a function of distance from the guiding interface $x_{2}=0$. It is assumed that both half-spaces of the waveguide are linear and lossless.

### 2.2. Elastic Drude-like Compliance $s_{44}^{\left(1\right)}\left(\omega \right)$ in the Metamaterial Half-Space ($x_{2}<0$)

The important assumption made throughout this paper is about the elastic compliance $s_{44}^{\left(1\right)}\left(\omega \right)$ in the metamaterial half-space ($x_{2}<0$). Namely, it is assumed that $s_{44}^{\left(1\right)}\left(\omega \right)$, as a function of angular frequency ω, is given explicitly by the following formula:

(1) $$s_{44}^{\left(1\right)}\left(\omega \right)=s_{0}\cdot \left(1-\frac{\omega_{p}^{2}}{\omega^{2}}\right)$$

where: $\omega_{p}$ is the angular frequency of the local mechanical resonances of the metamaterial and $s_{0}$ is its reference elastic compliance for ω→∞.

It is not difficult to notice that the elastic compliance $s_{44}^{\left(1\right)}\left(\omega \right)$ given by Equation (1), is formally identical to the dielectric function ε(ω) in Drude’s model of metals [18], in which the angular frequency $\omega_{p}$ is named the angular frequency of bulk plasma resonance [19].

Similarly, the density $\rho_{1}$ of the metamaterial half-space ($x_{2}<0$) corresponds to the magnetic permeability μ in Drude’s model of metals.

The second elastic half-space ($x_{2}<0$) is a conventional elastic material with a positive compliance $s_{44}^{\left(2\right)}>0$ and density $\rho_{2}>0$ that are both frequency independent.

In the following of this paper, it is assumed that the elastic compliance $s_{44}^{\left(1\right)}\left(\omega \right)$ in the metamaterial half-space ($x_{2}<0$) is given by Equation (1), which is an exact analogue to the dielectric function ε(ω) in Drude’s model of metals. This assumption not only simplifies further analysis but also provides us with a full analogy with the SPP electromagnetic waves propagating at a metal–dielectric interface. Therefore, the results obtained in the SPP domain may be almost automatically transferred to the SH elastic domain and vice versa.

### 2.3. Quantitative Model of the Elastic Metamaterial with a Drude-like Elastic Compliance

To develop a quantitative model for elastic metamaterials with the Drude-like elastic compliance $s_{44}^{\left(1\right)}\left(\omega \right)$, described by Equation (1), we will consider a number of electromechanical analogies based on the close affinity between the new SH elastic surface waves and the SPP electromagnetic modes propagating at a metal–dielectric interface.

The correspondence between the new SH elastic surface waves and the SPP electromagnetic waves stems from the fact that they share formally identical mathematical models, derived from the first physical principles. Namely, from the equations of motion (second Newton’s law) governing the behavior of an elastic continuum with parameters $s_{44}$ and ρ and Maxwell’s electromagnetic equations determining behavior of an electromagnetic continuum with parameters ε and μ.

The correspondence between the dielectric permeability and magnetic permeability and shear modulus and density can be expressed as follows: $\varepsilon \Leftrightarrow s_{44}$ and μ⇔ρ. In Section 4, we compare the properties of the new SH elastic surface waves and electromagnetic surface waves of the SPP type.

Consequently, the mathematical formulas that we can prove in the domain of the SPP electromagnetic waves using ε and μ can be automatically transferred to the domain of the new SH elastic surface waves, which employs $s_{44}$ and ρ.

We begin our analysis by proposing a one-dimensional model of a mechanical resonator with the elastic properties described by the equation analogous to the dielectric function ε(ω) in Drude’s model of metals.

**Figure 2:**
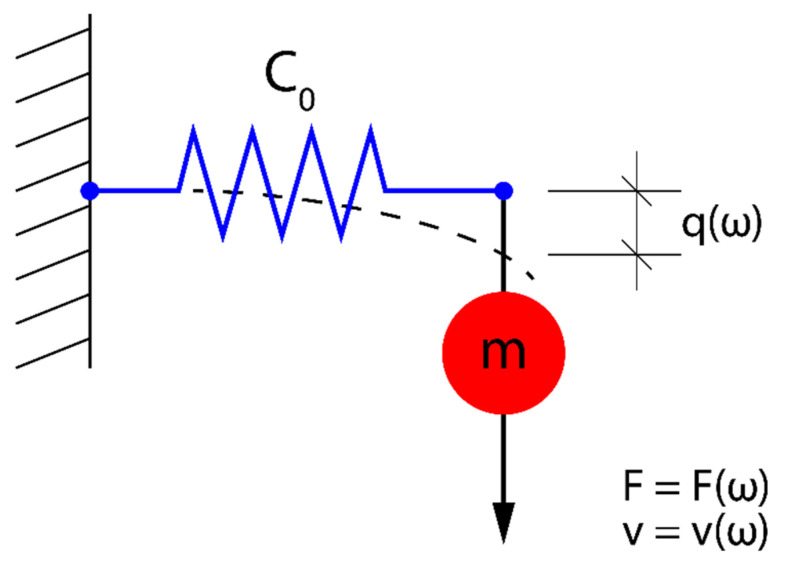
Spring-mass model of a mechanical resonator, whose effective shear elastic constant $C_{eff}\left(\omega \right)$, as a function of the angular frequency ω, is formally identical to the dielectric function ε(ω) in Drude’s model of metals. F(ω), q(ω) and v(ω)=jωq(ω) correspond, respectively, to the mechanical force, mechanical displacement, and acoustic velocity.

It is assumed that the one-dimensional mechanical resonator shown in Figure 2 performs shear vibrations and consists of an elastic spring with a compliance $C_{0}$ connected in series with mass m.

#### 2.3.1. Equivalent Circuit Representation of the Mechanical Resonator Shown in Figure 2

**Figure 3:**
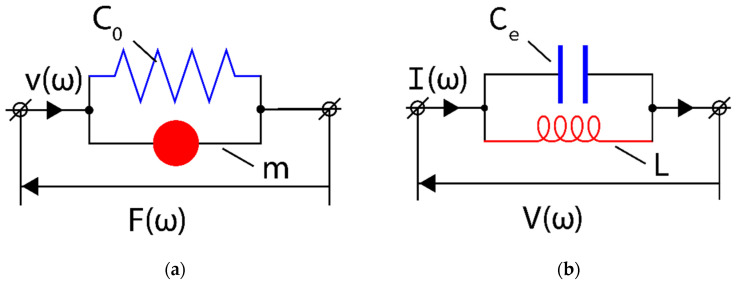
Mechanical (**a**) and electrical (**b**) equivalent circuits of the mechanical resonator presented in Figure 2. v(ω), F(ω), $C_{0}$ and m represent, respectively, the acoustic velocity, mechanical force, and elastic compliance of the spring and mass. Similarly, I(ω), V(ω), $C_{e}$ and L represent, respectively, the electric current, voltage, capacitance, and inductance.

The mechanical resonator given in Figure 2 can be represented by equivalent mechanical and electrical circuits with lumped elements $C_{0}$ and m (Figure 3a) and $C_{e}$ and L (Figure 3b).

The mechanical equivalent circuit shown in Figure 3a is governed by the equation of motion resulting from Newton’s second law of dynamics. However, the mechanical equivalent circuit shown in Figure 3a has its electric counterpart in the domain of electric circuits (see Figure 3b). Consequently, in the analysis of the mechanical equivalent circuit (Figure 3a) we can employ the methods and notions already developed in the theory of electric circuits, such as e.g., impedance or admittance. In particular, the mechanical admittance of the mechanical equivalent circuit, defined in the frequency ω domain as Y(ω)=v(ω)/F(ω), can be written as:

(2) $$Y\left(\omega \right)=j\omega C_{0}+\frac{1}{j\omega m}=j\omega C_{0}\left(1-\frac{\omega_{0}^{2}}{\omega^{2}}\right)$$

where $\omega_{0}=1/\sqrt{mC_{0}}$ is the resonant frequency of the mechanical resonator.

**Figure 4:**
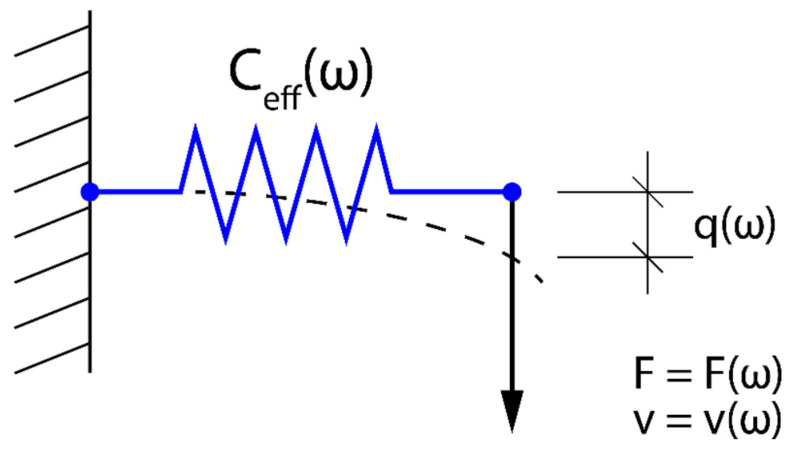
Equivalent lumped elastic compliance $C_{eff}\left(\omega \right)$ representing an overall behavior of the mechanical resonator from Figure 2.

Equation (2) shows that the overall behaviour of the mechanical resonator shown in Figure 2 can be expressed in terms of a resulting shear compliance $C_{eff}\left(\omega \right)$ represented by a lumped element (spring) in Figure 4.

By virtue of Equation (2), the equivalent lumped elastic compliance $C_{eff}\left(\omega \right)$ shown in Figure 4 is given by the following formula:

(3) $$C_{eff}=C_{0}\left(1-\frac{\omega_{0}^{2}}{\omega^{2}}\right)$$

The effective lumped (shear) elastic compliance $C_{eff}\left(\omega \right)$ is negative in the frequency range ($0-\omega_{0})$, in which it grows monotonically from −∞ to 0. It means that the mechanical velocity v(ω) lags in phase with respect to the driving mechanical force F(ω) by 180°.

Comparing Equation (3) with Equation (1), it is clear that the effective shear elastic compliance $C_{eff}\left(\omega \right)$ of the discrete representation of the mechanical resonator shown in Figure 2 and the elastic compliance $s_{44}^{\left(1\right)}\left(\omega \right)$ of the metamaterial elastic continuum ($x_{2}<0$) given by Equation (1) (Drude’s model) share the same frequency dependence, if $\omega_{0}$ is replaced by $\omega_{p}$. This is a very encouraging result since we are now in a position to propose an elementary cell (local oscillator) which constitutes the basis (microstructure) for the design of the elastic metamaterial continuum with a Drude-like elastic compliance $s_{44}^{\left(1\right)}\left(\omega \right)$, described by Equation (1).

In the development of a quantitative model of the elastic continuum with a Drude-like elastic compliance $s_{44}^{\left(1\right)}\left(\omega \right)$, it is prerequisite to identify the elementary cell of local oscillators embedded in the considered elastic host continuum.

#### 2.3.2. Unit Cell of Local Mechanical Resonators with SH Polarization

**Figure 5:**
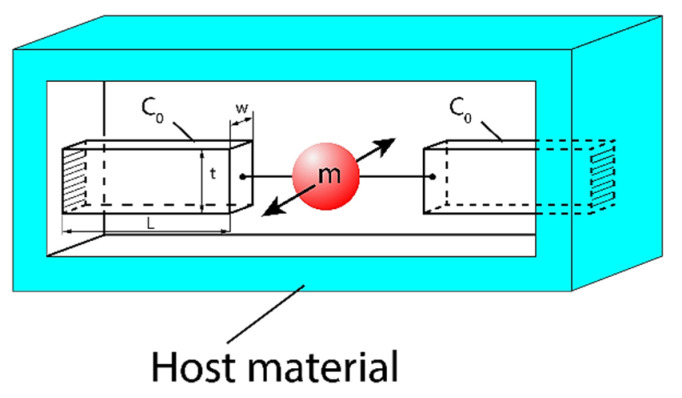
Proposed physical model of a local mechanical resonator with SH polarization embedded in an elastic host material.

As a unit cell that can be used as a local resonator, we choose the following structure, see Figure 5:

The proposed local resonator, embedded in a host elastic material, consists of a sphere of mass m connected to two microcantilevers, which act as a spring with an effective compliance $C_{0}/2$. It is assumed that the local resonator can vibrate only along the SH direction perpendicular to the line connecting the mass m with the cantilevers and perpendicular to the plane of Figure 5. As a result, the proposed local resonator can interact only with an SH wave propagating in the host material.

The elastic compliance of the microcantilever is given by the following formula: $C_{0}=4L^{3}/Ywt^{3}$, where L, w, t and Y stand, respectively, for the length, width, height, and Young’s modulus of the considered microcantilever. Consequently, the resonant frequency of the proposed local resonator equals $\omega_{0}=\sqrt{2/mC_{0}}$.

#### 2.3.3. Elastic Continuum with a Drude-like Elastic Compliance

The analytical formula for the average mechanical energy $W_{M}\left(\omega \right)$ stored in the mechanical resonator represented by the discrete mechanical circuit shown in Figure 3a equals:

(4) $$W_{M}\left(\omega \right)=\frac{1}{4}\left(1+\frac{\omega_{0}^{2}}{\omega^{2}}\right)C_{0}{\left|F\right|}^{2}$$

Up to now, we are still in the domain of the lumped element circuit theory. However, we are going now to perform the first crucial step by transferring the results obtained in the discrete 1-D circuit domain to the 3-D domain of the metamaterial continuum.

**Figure 6:**
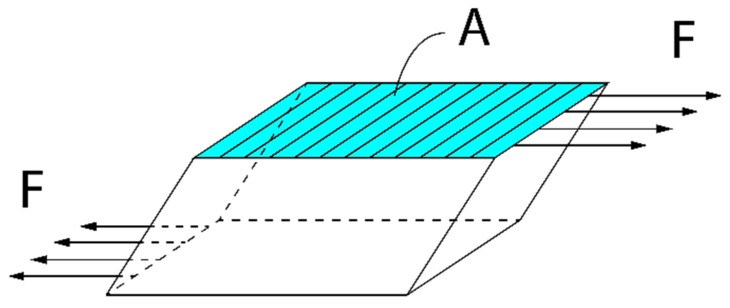
Schematic representation of an elementary shear resonator. The shear force F is acting on the appropriate surface A.

Indeed, in analogy to Equation (4) we are in a position to show that the average mechanical energy density $w_{M}\left(\omega \right)$ stored in the corresponding elastic continuum equals:

(5) $$w_{M}\left(\omega \right)=\frac{1}{4}\left(1+\frac{\omega_{0}^{2}}{\omega^{2}}\right)s_{0}{\left|\tau_{23}\right|}^{2}$$

where: $s_{0}$ is the elastic compliance of the corresponding elastic continuum, $\tau_{23}$ is the shear stress equal to $\tau_{23}=F/A$ and F is the shear force acting on the surface A of the local oscillator, see Figure 6.

**Figure 7:**
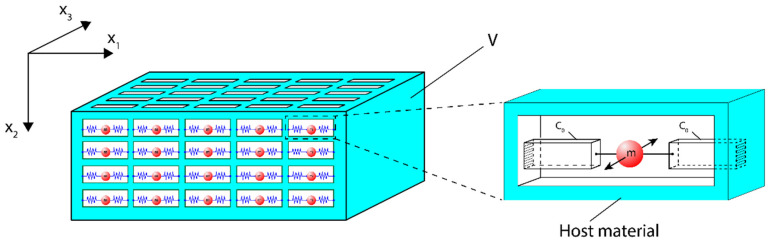
A model of an elastic metamaterial with a Drude-like dependence of elastic compliance $s_{44}^{\left(1\right)}\left(\omega \right)$ on the angular frequency ω. A set of n local mechanical oscillators is embedded into the host continuum material in the reference volume V. The snippet on the right side shows details of the local resonator presented in more detail in Section 2.3.2.

Therefore, the mechanical energy $W_{M}$ stored in the reference volume V (shown in Figure 7) in the elastic metamaterial equals:

(6) $$W_{M}\left(\omega \right)=\frac{1}{4}\left(1+\frac{\omega_{0}^{2}}{\omega^{2}}\right)s_{0}^{eff}{\left|\tau_{23}\right|}^{2}V=\frac{1}{4}\left(1+\frac{\omega_{0}^{2}}{\omega^{2}}\right)n\cdot C_{0}{\left|F\right|}^{2}$$

where: n is the number of local shear resonators contained in the reference volume V (see Figure 7). The coefficient ${\left(s_{0}\right)}^{eff}$ in Equation (6) represents the average value of the elastic compliance of the resulting 3-D elastic metamaterial continuum.

Now we are going to perform the second crucial step in our development of the quantitative model of the elastic continuum with a Drude-like elastic compliance. This time, we will use the equation developed in the electromagnetic domain by V.L. Ginzburg in [20] for the energy density of the electromagnetic continuum, whose material parameters are dispersive, i.e., they change with the angular frequency ω.

Indeed, using Equation (5) and transferring the electromagnetic equation B.2.5 from reference [20] into the domain of elastodynamics we obtain:

(7) $$\frac{d}{d\omega }\left(\omega \frac{s_{44}\left(\omega \right)}{s_{0}^{eff}}\right)=\left(1+\frac{\omega_{0}^{2}}{\omega^{2}}\right)$$

In the derivation of Equation (7) we employed the correspondence between the dielectric function ε(ω) and elastic compliance $s_{44}\left(\omega \right)$, shown in Section 4.

At this moment we are almost done. To obtain a quantitative model of the elastic continuum with a Drude-like elastic compliance we need to perform only a few technical steps. At first, we will integrate Equation (7) over ω arriving at the following formula:

(8) $$\frac{s_{44}\left(\omega \right)}{s_{0}^{eff}}=\left(1-\frac{\omega_{0}^{2}}{\omega^{2}}\right)$$

It is not difficult to note that Equation (8) is exactly Drude’s relation describing the elastic compliance $s_{44}\left(\omega \right)$ of the resulting elastic metamaterial continuum as a function of angular frequency ω (see Equation (1) in Section 2.1).

In the last technical step, we must relate the averaged value of the effective elastic compliance $s_{0}^{eff}$ of the resultant elastic metamaterial continuum with the parameters of embedded elementary resonators in an elastic host material.

In fact, since the shear stress $\left|\tau_{23}\right|$, acting on an elementary resonator with the surface A (see Figure 6), equals $\left|\tau_{23}\right|=\left|F\right|/A$, by virtue of Equation (6) we can write the following:

(9) $$s_{0}^{eff}=\frac{nC_{0}{\left|F\right|}^{2}}{{\left|\tau_{23}\right|}^{2}V}=\frac{nC_{0}A^{2}}{V}$$

Now we have all the necessary elements to present our final model of an elastic metamaterial with a Drude-like elastic compliance $s_{44}\left(\omega \right)$, see Figure 7 below.

### 2.4. Fabrication of the Elastic Metamaterial with a Drude-like Elastic Compliance $s_{44}\left(\omega \right)$

Elastic metamaterial with a Drude-like elastic compliance in a certain frequency range was already proposed in [21]. The unit cell of the proposed metamaterial was composed of four tungsten rods with four adjacent vacuum cavities embedded in a host foam. A circular vacuum cavity was placed in the center of the unit cell. Negative elastic compliance was due to the quadrupolar resonance occurring in the unit cell The negativity of the elastic compliance $s_{44}^{\left(1\right)}\left(\omega \right)$ was confirmed by the corresponding FEM calculations. The elastic compliance of the metamaterial had some characteristics of Drude’s model but was by no means described by the analytical formula given by Equation (1).

**Figure 8:**
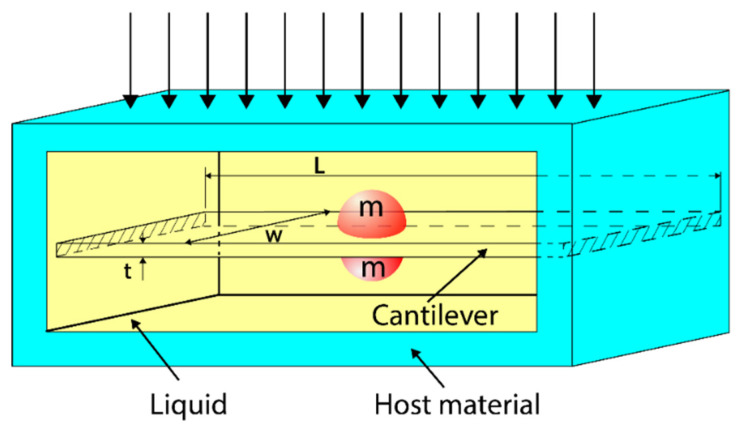
Practical realization of the proposed local mechanical resonator with SH polarization embedded in an elastic host material.

In the following, we have included numerical data for material parameters of the elementary mechanical oscillator shown in Figure 8 as well as the resulting resonant frequency $f_{0}$ and effective mechanical compliance ${\left(s_{0}\right)}^{eff}$.

Numerical example:

As a unit cell that can be used as a local resonator, we can choose the following structure, see Figure 8:

Effective elastic compliance $C_{0}$ of the cantilever shown in Figure 8, treated as a spring, can be expressed as: $C_{0}=4L^{3}/Ywt^{3}$: where: L = length, w = width, t = height and Y = Young’s modulus.

Material parameters of the cantilever shown in Figure 8 were chosen as follows:

L=5 mm, w=3 mm, t=1 mm and Y=100 GPa: (Bronze).

Reference Volume V was assumed as: $=2\times 10^{-5}{\;m}^{3}$.

Surface A of the elementary shear resonator from Figure 6 equals $A=10\;{\mathrm{mm}}^{2}$.

The number of local resonators n in the reference volume V is equal to n=200.

The mass of the sphere is: $=10^{-5}\;\mathrm{kg}$: (Tin-lead alloy).

Employing the above set of parameters, we get: $C_{0}=\frac{1}{8}10^{-5}\;\left[\frac{m}{N}\right]$; $A^{2}/V=\frac{1}{2}10^{-5}\;\left[m\right]$.

The resonant frequency of the local resonator amounts to $f_{0}=\frac{1}{2\pi }\cdot \sqrt{2/mC_{0}}\approx 21\;\mathrm{kHz}$. As a host material, we can choose one of the plastics, for example: Nylon PA-6.

Finally, the effective elastic compliance equals: ${\left(s_{0}\right)}^{eff}=n\cdot C_{0}\cdot A^{2}/V\approx 6\cdot 10^{-10}\;\left[m^{2}/N\right]$.

Ultrasonic waves in the considered frequency range (e.g., 50 KHz) can be generated and received using standard ultrasonic transducers operating in a conventional experimental setup consisting of a pulser-receiver, a measuring head with ultrasonic transducers, and a control electronic unit (PC computer).

The velocity of ultrasonic waves can be determined, using the above experimental setup, from measurements of the time-of-flight (TOF) between the selected ultrasonic impulses. In the precise determination of the time of flight and therefore the velocity of ultrasonic waves, we can employ the cross-correlation method, which can be effectively implemented digitally within the controlling PC computer.

It should be noted that the new SH elastic surface waves can also propagate in another class of elastic waveguides, in which the elastic compliance $s_{44}^{\left(1\right)}\left(\omega \right)$ of the metamaterial half-space is described by an analytical formula different that the Drude’s formula, given by Equation (1). Namely, the analysis performed in the submitted manuscript will be also valid (after some modifications) when the elastic compliance $s_{44}^{\left(1\right)}\left(\omega \right)$ fulfils the following 2 conditions:

1. elastic compliance $s_{44}^{\left(1\right)}\left(\omega \right)$ is negative and increases monotonically in the frequency range $\omega_{1}<\omega <\omega_{2}$,

and

2. elastic compliance $s_{44}^{\left(1\right)}\left(\omega \right)$ equals zero for the frequency $\omega =\omega_{2}$.

As an example of the elastic compliance $s_{44}^{\left(1\right)}\left(\omega \right)$ that satisfies the above two conditions we can invoke a Lorentz-like function implying the following formula: $s_{44}^{\left(1\right)}\left(\omega \right)=s_{0}\left(1-\frac{\omega_{0}^{2}}{\omega^{2}-\omega_{1}^{2}}\right)$. All analytical equations developed in the submitted manuscript will be valid (after some modifications) for the Lorentz-like elastic compliance $s_{44}^{\left(1\right)}\left(\omega \right)$. Similar can be said about figures presented in Section 5 which will be different, but they will preserve anyway their qualitative properties. However, besides some complications the Lorentz-like elastic compliance does not bring important new phenomena, which are not already present in the Drude-like model.

Therefore, for the sake of simplicity and possible comparison with the SPP electromagnetic waves, which are commonly analyzed with the dielectric function ε(ω) of the Drude type, in the submitted manuscript we assumed that the elastic compliance $s_{44}^{\left(1\right)}\left(\omega \right)$ in the metamaterial half-space is described by the Drude-like Equation (1).

The elastic metamaterial with a Drude-like elastic compliance, described by Equation (1) may be fabricated using 3-D printers and dip-in direct-laser-writing optical lithography [22]. This activity will be the subject of the author’s future works.

## 3. Mathematical Model

### 3.1. Mechanical Displacement $u_{3}^{\left(i\right)}\left(x_{2}\right)$ and Stresses $\tau_{23}^{\left(i\right)}\left(x_{2}\right),\;\tau_{13}^{\left(i\right)}\left(x_{2}\right)$

Since new SH elastic surface waves are time-harmonic, propagate in the direction $x_{1}$ and are uniform along the transverse direction $x_{3}$, their mechanical displacement $u_{3}^{\left(i\right)}$, in both half-spaces (i=1,2) shown in Figure 1, will be sought in the following generic form:

(10) $$u_{3}^{\left(i\right)}=u_{3}^{\left(i\right)}\left(x_{2}\right)exp\left[j\left(k\cdot x_{1}-\omega t\right)\right]$$

where $u_{3}^{\left(i\right)}\left(x_{2}\right)$ expresses variations of the mechanical displacement in the transverse direction $x_{2}$, k is the wavenumber of the new SH elastic surface wave and ω its angular frequency.

The mechanical displacement $u_{3}^{\left(i\right)}$ in both half-spaces of the waveguide is governed by the wave equation, resulting from the second Newton’s law, which with the help of Equation (10) reduces to the second order ordinary differential equation of the Helmholtz type [23]:

(11) $$\left[\frac{d^{2}}{dx_{2}}+k_{i}^{2}\right]\cdot u_{3}^{\left(i\right)}\left(x_{2}\right)=k^{2}\cdot u_{3}^{\left(i\right)}\left(x_{2}\right)$$

where $k_{i}=\omega /v_{i}$ is the wavenumber of SH bulk waves in both elastic half-spaces number i=1, 2. In the conventional elastic half-space (i=2) the wavenumber $k_{2}^{2}=\omega^{2}s_{44}^{\left(2\right)}\rho_{2}$ is positive and in the metamaterial half-space (i=1) the wavenumber $k_{1}^{2}=-\omega^{2}\left|s_{44}^{\left(1\right)}\right|\rho_{1}$ is always negative in the angular frequency range $0<\omega \leq \omega_{p}$.

Since the mechanical displacement $u_{3}^{\left(i\right)}\left(x_{2}\right)$ of the new SH elastic surface wave must vanish at large distances from the guiding interface $x_{2}=0$, namely for $x_{2}\to \pm \infty$, the solution of the Helmholtz Equation (11) will be sought in the following form:

(12) $$u_{3}^{\left(i\right)}\left(x_{2}\right)=C_{i}e^{\pm q_{i}x_{2}}$$

where $C_{i}$ (i=1,2) are arbitrary amplitude coefficients and the transverse wave numbers $q_{i}$ are real (waveguide is lossless) and according to the Helmholtz Equation (11) is given by $q_{i}=\sqrt{\left(k^{2}-k_{i}^{2}\right)}$, where $k_{i}=\omega \sqrt{s_{44}^{\left(i\right)}\rho_{i}}$ are wavenumbers of bulk SH waves in the metamaterial half-space $x_{2}<0$ (i=1) and conventional elastic half-space $x_{2}\geq 0$ (i=2).

In the following of this paper, we will use two shear stresses of the new SH elastic surface wave, namely $\tau_{23}^{\left(i\right)}$ and $\tau_{13}^{\left(i\right)}$ that are defined, respectively, as:

$$\tau_{23}^{\left(i\right)}=\left(1/s_{44}^{\left(i\right)}\right)\partial u_{3}^{\left(i\right)}/\partial x_{2}\;\mathrm{and}\;\tau_{13}^{\left(i\right)}=\left(1/s_{44}^{\left(i\right)}\right)\partial u_{3}^{\left(i\right)}/\partial x_{1}.$$

Consequently, we can write the following formulas:

(13) $$u_{3}^{\left(i\right)}\left(x_{2}\right)=C_{i}\cdot exp\left(\pm q_{i}x_{2}\right)$$

(14) $$\tau_{23}^{\left(i\right)}\left(x_{2}\right)=\frac{1}{s_{44}^{\left(i\right)}}C_{i}\cdot \left(\pm q_{i}\right)\cdot exp\left(\pm q_{i}x_{2}\right)$$

(15) $$\tau_{13}^{\left(i\right)}\left(x_{2}\right)=\frac{1}{s_{44}^{\left(i\right)}}C_{i}\cdot jk\cdot exp\left(\pm q_{i}x_{2}\right)$$

(16) $$\tau_{23}^{\left(i\right)}\left(x_{2}\right)=\frac{1}{s_{44}^{\left(i\right)}}C_{i}\cdot \left(\pm q_{i}\right)\cdot exp\left(\pm q_{i}x_{2}\right)$$

where the index i=1,2.

To provide an exponential decay of $u_{3}^{\left(i\right)}(x_{2}),\;\tau_{23}^{\left(i\right)}(x_{2})$ and $\tau_{13}^{\left(i\right)}(x_{2})$ the transverse wavenumber $q_{i}$ in Equations (13)–(16) have to be preceded by sign − in the convention elastic half-space ($x_{2}\geq 0$) and by sign + in the metamaterial half-space ($x_{2}<0$), since $q_{i}$ (i=1,2) in Equations (13)–(16) are real and positive.

### 3.2. Boundary Conditions and Dispersion Equation

From physical considerations it is obvious that the mechanical displacement $u_{3}^{\left(i\right)}\left(x_{2}\right)$ and the shear stress $\tau_{23}^{\left(i\right)}\left(x_{2}\right)$ must be continuous at the interface $x_{2}=0$, namely:

(17) $$u_{3}^{\left(1\right)}\left(x_{2}=0\right)=u_{3}^{\left(2\right)}\left(x_{2}=0\right)$$

(18) $$\tau_{23}^{\left(1\right)}\left(x_{2}=0\right)=\tau_{23}^{\left(2\right)}\left(x_{2}=0\right)$$

Substituting Equations (13) and (14) into boundary conditions, Equations (17) and (18), one obtains two linear homogeneous algebraic equations for two unknown amplitude coefficients $C_{1}$ and $C_{2}$, namely:

(19) $$C_{1}=C_{2}$$

(20) $$C_{1}\frac{q_{1}}{s_{44}^{\left(1\right)}\left(\omega \right)}=-C_{2}\;\frac{q_{2}}{s_{44}^{\left(2\right)}}$$

Combining Equations (19) and (20), we get the following dispersion equation for the new SH elastic surface waves:

(21) $$\frac{q_{1}}{-s_{44}^{\left(1\right)}\left(\omega \right)}=\frac{q_{2}}{s_{44}^{\left(2\right)}}$$

The sign “−” before the compliance $-s_{44}^{\left(1\right)}\left(\omega \right)$ plays a crucial role in the analysis of new SH elastic surface waves, since it implies that if the transverse wavenumbers $q_{1}$ and $q_{2}$ are positive, the elastic compliances $s_{44}^{\left(1\right)}\left(\omega \right)$, $s_{44}^{\left(2\right)}$ must be of the opposite sign $s_{44}^{\left(1\right)}\left(\omega \right)\cdot s_{44}^{\left(2\right)}<0$. Consequently, if the elastic compliance $s_{44}^{\left(1\right)}\left(\omega \right)$ (see Equation (1)) in the metamaterial half-space is negative for $\omega <\omega_{p}$, the compliance $s_{44}^{\left(2\right)}$ have to be positive (see Figure 1).

Since $C_{1}=C_{2}$ (see Equation (19)) in the following of this paper we will use only one amplitude coefficient, denoted as $C=C_{1}=C_{2}$.

### 3.3. Wavenumber k(ω)

Substituting Equation (16), for transverse wavenumbers $q_{1}$ and $q_{2}$, in the dispersion relation Equation (21), one obtains the following formula for the wavenumber k(ω) of the new SH elastic surface wave:

(22) $$k\left(\omega \right)=k_{2}\sqrt{\frac{s_{44}^{\left(1\right)}\left(\omega \right)}{s_{44}^{\left(1\right)}\left(\omega \right)+s_{44}^{\left(2\right)}}}\;\sqrt{\frac{s_{44}^{\left(2\right)}\frac{\rho_{1}}{\rho_{2}}-s_{44}^{\left(1\right)}\left(\omega \right)}{s_{44}^{\left(2\right)}-s_{44}^{\left(1\right)}\left(\omega \right)}}$$

where the wavenumber of bulk SH waves in the conventional elastic half-space $k_{2}=\omega \sqrt{s_{44}^{\left(2\right)}\rho_{2}}$.

Since the wavenumber k(ω) of the new SH elastic surface wave must be real and positive, Equation (22) imposes the following two necessary conditions on $s_{44}^{\left(1\right)}\left(\omega \right)$ and $s_{44}^{\left(2\right)}$:

(23) $$\left(s_{44}^{\left(1\right)}\left(\omega \right)<0\right)\;\mathrm{and}\;\left(s_{44}^{\left(1\right)}\left(\omega \right)+s_{44}^{\left(2\right)}\right)<0$$

The first condition requires that $\omega <\omega_{p}$ and the second gives rise to $\omega <\omega_{sp}$, where the cut-off angular frequency $\omega_{sp}$ and the angular frequency of local resonances $\omega_{p}$ are related by:

(24) $$\omega_{sp}=\omega_{p}/\sqrt{\frac{s_{44}^{\left(2\right)}}{s_{0}}+1}$$

Since $\omega_{p}$ is always higher than $\omega_{sp}$ ($\omega_{p}>\omega_{sp}$), the two conditions given by Equation (23) imply that the frequency ω of the new SH elastic surface wave must be limited to the range $0<\omega <\omega_{sp}$.

In the context of the SPP electromagnetic surface waves, the angular frequency $\omega_{sp}$ is called the surface plasmon resonance frequency [19].

### 3.4. Phase Velocity $v_{p}\left(\omega \right)$

Since by definition $k\left(\omega \right)=\omega /v_{p}\left(\omega \right)$, the analytical formula for the phase velocity $v_{p}\left(\omega \right)$ of new SH elastic surface waves results immediately from Equation (22):

(25) $$v_{p}\left(\omega \right)=v_{2}\sqrt{\frac{s_{44}^{\left(1\right)}\left(\omega \right)+s_{44}^{\left(2\right)}}{s_{44}^{\left(1\right)}\left(\omega \right)}}\;\sqrt{\frac{s_{44}^{\left(2\right)}-s_{44}^{\left(1\right)}\left(\omega \right)}{s_{44}^{\left(2\right)}\frac{\rho_{1}}{\rho_{2}}-s_{44}^{\left(1\right)}\left(\omega \right)}}$$

where $v_{2}=1/\sqrt{s_{44}^{\left(2\right)}\rho_{2}}$ is the phase velocity of bulk SH waves in the conventional elastic half-space.

### 3.5. Group Velocity $v_{g}\left(\omega \right)$

Differentiation of Equation (22) for the wavenumber k(ω), with respect to the angular frequency ω, leads to the following formula for the group velocity $v_{g}\left(\omega \right)=d\omega /dk$ of the new SH surface wave:

(26) $$\begin{array}{c} \frac{v_{g}\left(\omega \right)}{v_{2}}\frac{v_{p}\left(\omega \right)}{v_{2}}=\\ \frac{{\left[{\left[s_{44}^{\left(2\right)}\right]}^{2}-{\left[s_{44}^{\left(1\right)}\left(\omega \right)\right]}^{2}\right]}^{2}}{s_{44}^{\left(1\right)}\left(\omega \right)\left[\frac{\rho_{1}}{\rho_{2}}s_{44}^{\left(2\right)}-s_{44}^{\left(1\right)}\left(\omega \right)\right]\left[{\left[s_{44}^{\left(2\right)}\right]}^{2}-{\left[s_{44}^{\left(1\right)}\left(\omega \right)\right]}^{2}\right]+\frac{\omega }{2}\frac{ds_{44}^{\left(1\right)}\left(\omega \right)}{d\omega }\left[\frac{\rho_{1}}{\rho_{2}}\left[{\left[s_{44}^{\left(2\right)}\right]}^{2}+{\left[s_{44}^{\left(1\right)}\left(\omega \right)\right]}^{2}\right]-2s_{44}^{\left(1\right)}\left(\omega \right)s_{44}^{\left(2\right)}\right]} \end{array}$$

Despite its relative complexity, Equation (26) is quite elementary and can be easily implemented in numerical calculations.

### 3.6. Penetration Depths $\delta_{1}\left(\omega \right),\delta_{2}\left(\omega \right)$ in Both Half-Spaces of the Waveguide

The penetration depth in the metamaterial half-space $x_{2}<0$ is defined as $\delta_{1}\left(\omega \right)=1/q_{1}\left(\omega \right)$, where the transverse wave number $q_{1}\left(\omega \right)=\sqrt{k^{2}-k_{1}^{2}}$ (see Equation (16)) and $k_{1}^{2}=\omega^{2}s_{44}^{\left(1\right)}\left(\omega \right)\rho_{1}$. Similarly, in the conventional elastic half-space $x_{2}\geq 0$ we have $\delta_{2}\left(\omega \right)=1/q_{2}\left(\omega \right)$, where the transverse wavenumber $q_{2}\left(\omega \right)=\sqrt{k^{2}-k_{2}^{2}}$ (see Equation (16)) and $k_{2}^{2}=\omega^{2}s_{44}^{\left(2\right)}\left(\omega \right)\rho_{2}$.

Consequently, substituting Equation (22) for the wavenumber k into Equation (16) for the transverse wavenumbers $q_{1}$ and $q_{2}$ and noting that λ=2π/k, one obtains:

(27) $$\delta_{1}\left(\omega \right)=\frac{\lambda }{2\pi }\sqrt{\frac{s_{44}^{\left(2\right)}\left[-s_{44}^{\left(1\right)}\left(\omega \right)+s_{44}^{\left(2\right)}\frac{\rho_{1}}{\rho_{2}}\right]}{-s_{44}^{\left(1\right)}\left(\omega \right)\left[s_{44}^{\left(2\right)}-s_{44}^{\left(1\right)}\left(\omega \right)\frac{\rho_{1}}{\rho_{2}}\right]}}$$

(28) $$\delta_{2}\left(\omega \right)=\frac{\lambda }{2\pi }\sqrt{\frac{-s_{44}^{\left(1\right)}\left(\omega \right)\left[-s_{44}^{\left(1\right)}\left(\omega \right)+s_{44}^{\left(2\right)}\frac{\rho_{1}}{\rho_{2}}\right]}{s_{44}^{\left(2\right)}\left[s_{44}^{\left(2\right)}-s_{44}^{\left(1\right)}\left(\omega \right)\frac{\rho_{1}}{\rho_{2}}\right]}}$$

where λ is the wavelength of the new SH elastic surface wave.

In general, the ratio of the penetration depths $\delta_{1}\left(\omega \right)$, $\delta_{2}\left(\omega \right)$ is expressed by the dispersion equation (Equation (21)), i.e., $\delta_{2}\left(\omega \right)/\delta_{1}\left(\omega \right)=-s_{44}^{\left(1\right)}\left(\omega \right)/s_{44}^{\left(2\right)}$ that is independent on $\rho_{1}/\rho_{2}$. On the other hand, by virtue of Equations (27) and (28), the product of the normalized penetration depths equals:

(29) $$\frac{\delta_{1}\left(\omega \right)}{\lambda }\cdot \frac{\delta_{2}\left(\omega \right)}{\lambda }={\left(\frac{1}{2\pi }\right)}^{2}\;\frac{-s_{44}^{\left(1\right)}\left(\omega \right)+s_{44}^{\left(2\right)}\frac{\rho_{1}}{\rho_{2}}}{s_{44}^{\left(2\right)}-s_{44}^{\left(1\right)}\left(\omega \right)\frac{\rho_{1}}{\rho_{2}}}$$

However, if the density in both half-spaces of the waveguide is the same ($\rho_{1}=\rho_{2}$) then Equation (29) reduces to:

(30) $$\frac{\delta_{1}\left(\omega \right)}{\lambda }\cdot \frac{\delta_{2}\left(\omega \right)}{\lambda }=\;{\left(\frac{1}{2\pi }\right)}^{2}$$

Thus, if the density in both half-spaces of the waveguide is identical ($\rho_{1}=\rho_{2}$) the product of the normalized penetration depths $\delta_{1}\left(\omega \right)\delta_{2}\left(\omega \right)/\lambda^{2}$ is independent of angular frequency ω and material constants of the waveguide and equals ${\left(1/2\pi \right)}^{2}\approx 0.025$. In other words, if $\rho_{1}=\rho_{2}$ both normalized penetration depths $\delta_{1}\left(\omega \right)/\lambda$, $\delta_{2}\left(\omega \right)/\lambda$ are inversely proportional. As a result, if $\delta_{1}\left(\omega \right)/\lambda$ increases then $\delta_{2}\left(\omega \right)/\lambda$ decreases accordingly to Equation (30) and vice versa. Simultaneously, if the angular frequency $\omega \to \omega_{sp}$ then both $\delta_{1}\left(\omega \right)/\lambda$ and $\delta_{2}\left(\omega \right)/\lambda$ are subwavelength and tend to the same value 1/2π.

### 3.7. Net Active Power Flow $P_{1}^{\left(1\right)}\left(\omega \right),P_{1}^{\left(2\right)}\left(\omega \right)$ in the Direction of Propagation $x_{1}$

The complex Poynting vector$\;P_{1}^{\left(i\right)}\left(x_{2}\right)$, in the direction of propagation $x_{1}$, of new SH elastic surface waves can be expressed as $P_{1}^{\left(i\right)}\left(x_{2}\right)=-\frac{1}{2}\left[\tau_{13}^{\left(i\right)}\left(x_{2}\right)\cdot {\left(-j\omega u_{3}^{\left(i\right)}\left(x_{2}\right)\;\right)}^{*}\right]$, where $u_{3}^{\left(i\right)}\left(x_{2}\right)$ is the mechanical displacement (Equation (5)) and $\tau_{13}^{\left(i\right)}\left(x_{2}\right)$ is the mechanical stress (Equation (15)), where i=1, 2.

Similarly, the net complex power flow (per unit length along the axis $x_{3}$) in the metamaterial half-space ($x_{2}<0$) is defined as $P_{1}^{\left(1\right)}\left(\omega \right)=\int_{-\infty }^{0}P_{1}^{\left(1\right)}\left(x_{2}\right)dx_{2}$ (see Figure 1) and in the conventional elastic half-space ($x_{2}\geq 0$) by $P_{1}^{\left(2\right)}\left(\omega \right)=\int_{0}^{\infty }P_{1}^{\left(2\right)}\left(x_{2}\right)dx_{2}$.

Consequently, using Equations (13) and (15), it can be shown that the net complex power flows $P_{1}^{\left(1\right)}(\omega )$ and $P_{1}^{\left(2\right)}(\omega )$ in both half-spaces of the waveguide are given by:

(31) $$P_{1}^{\left(1\right)}(\omega )=-\frac{1}{4}{\left|C\right|}^{2}\frac{k(\omega )\omega }{-s_{44}^{\left(1\right)}(\omega )q_{1}(\omega )}$$

(32) $$P_{1}^{\left(2\right)}(\omega )=\frac{1}{4}{\left|C\right|}^{2}\frac{k(\omega )\omega }{s_{44}^{\left(2\right)}q_{2}(\omega )}$$

where C is an arbitrary amplitude coefficient.

It should be noticed that all field variables entering Equations (31) and (32) are real. Therefore, the power flows $P_{1}^{\left(1\right)}\left(\omega \right)$ and $P_{1}^{\left(2\right)}\left(\omega \right)$ in both half-spaces of the waveguide are active. In other words, new SH elastic surface waves can effectively transfer the active power along the guiding interface $x_{2}=0$ in the direction of propagation $x_{1}$.

Employing the dispersion Equation (21) in conjunction with Equations (31) and (32), the ratio of the net active powers flows $P_{1}^{\left(1\right)}\left(\omega \right)/P_{1}^{\left(2\right)}\left(\omega \right)$ in both half-spaces of the waveguide is given by the following:

(33) $$\frac{P_{1}^{\left(1\right)}\left(\omega \right)}{P_{1}^{\left(2\right)}\left(\omega \right)}=\frac{s_{44}^{\left(2\right)}}{s_{44}^{\left(1\right)}\left(\omega \right)}\frac{q_{2}\left(\omega \right)}{q_{1}\left(\omega \right)}=-{\left[\frac{\delta_{1}\left(\omega \right)}{\delta_{2}\left(\omega \right)}\right]}^{2}$$

Note that the ratio of the net active power flows in both half-spaces is always negative, since $s_{44}^{\left(1\right)}\left(\omega \right)$ and $s_{44}^{\left(2\right)}$ are of the opposite sign and the transverse wavenumbers are real and positive $q_{1}\left(\omega \right),\;\;q_{2}\left(\omega \right)>0$. Consequently, $P_{1}^{\left(1\right)}\left(\omega \right)$ and $P_{1}^{\left(2\right)}\left(\omega \right)$ propagate in opposite directions along axis $x_{1}$.

### 3.8. Average Reactive Power Flow $P_{2}^{\left(1\right)}\left(\omega \right),P_{2}^{\left(2\right)}\left(\omega \right)$ in the Transverse Direction $x_{2}$

The complex Poynting vector$\;P_{2}^{\left(i\right)}\left(x_{2}\right)$, in the transverse direction $x_{2}$, of new SH elastic surface waves can be expressed as $P_{2}^{\left(i\right)}\left(x_{2}\right)=-\frac{1}{2}\left[\tau_{23}^{\left(i\right)}\left(x_{2}\right)\cdot {\left(-j\omega u_{3}^{\left(i\right)}\left(x_{2}\right)\;\right)}^{*}\right]$, where $u_{3}^{\left(i\right)}\left(x_{2}\right)$ is the mechanical displacement (Equation (13)) and $\tau_{23}^{\left(i\right)}\left(x_{2}\right)$ is the mechanical stress (Equation (14)), where i=1, 2.

Similarly, the average complex power flow (per unit length along the axis $x_{3}$) in the metamaterial half-space ($x_{2}<0$) is defined as $P_{2}^{\left(1\right)}\left(\omega \right)=\int_{-\infty }^{0}P_{2}^{\left(1\right)}\left(x_{2}\right)dx_{2}$ (see Figure 1) and in the conventional elastic half-space ($x_{2}\geq 0$) by $P_{2}^{\left(2\right)}\left(\omega \right)=\int_{0}^{\infty }P_{2}^{\left(2\right)}\left(x_{2}\right)dx_{2}$.

Consequently, using Equations (13) and (14) it can be shown that the average complex power flow $P_{2}^{\left(1\right)}\left(\omega \right)$ and $P_{2}^{\left(2\right)}\left(\omega \right)$ in both half-spaces are given by:

(34) $$P_{2}^{\left(1\right)}\left(\omega \right)=+j\frac{\omega }{4}{\left|C\right|}^{2}\frac{1}{-s_{44}^{\left(1\right)}\left(\omega \right)}$$

(35) $$P_{2}^{\left(2\right)}\left(\omega \right)=+j\frac{\omega }{4}{\left|C\right|}^{2}\frac{1}{s_{44}^{\left(2\right)}}$$

Thus, if ω→0 then $P_{2}^{\left(1\right)}\left(\omega \right)$ and $P_{2}^{\left(2\right)}\left(\omega \right)$ both tend to zero. On the other hand, if $\omega \to \omega_{sp}$ then $P_{2}^{\left(2\right)}\left(\omega \right)$ and $P_{2}^{\left(1\right)}\left(\omega \right)$ tend to the same value, namely $j\left(\omega_{sp}/4\right){\left|C\right|}^{2}/s_{44}^{\left(2\right)}$.

Since the elastic compliance $s_{44}^{\left(1\right)}\left(\omega \right)$ is negative, in the frequency range $0<\omega <\omega_{sp}$, the average reactive power flows $P_{2}^{\left(1\right)}\left(\omega \right),\;P_{2}^{\left(2\right)}\left(\omega \right)$, in both half-spaces, are both positive (+) and correspond to the inductive type of the reactive power, in analogy to SPP electromagnetic waves.

Using Equation (1) together with Equations (34) and (35), the ratio of the average reactive power flows in both half-spaces can be written as:

(36) $$\frac{P_{2}^{\left(1\right)}\left(\omega \right)}{P_{2}^{\left(2\right)}\left(\omega \right)}=-\frac{s_{44}^{\left(2\right)}}{s_{44}^{\left(1\right)}\left(\omega \right)}=\frac{\delta_{1}\left(\omega \right)}{\delta_{2}\left(\omega \right)}$$

Comparing Equations (33) and (36), one obtains a rather unexpected relation between the net active power flows $P_{1}^{\left(1\right)}\left(\omega \right),P_{1}^{\left(2\right)}\left(\omega \right)$ in the direction of propagation $x_{1}$ and the average reactive power flows $P_{2}^{\left(1\right)}\left(\omega \right),P_{2}^{\left(2\right)}\left(\omega \right)$ in the transverse direction $x_{2}$, namely:

(37) $$\frac{P_{1}^{\left(1\right)}\left(\omega \right)}{P_{1}^{\left(2\right)}\left(\omega \right)}=-{\left[\frac{P_{2}^{\left(1\right)}\left(\omega \right)}{P_{2}^{\left(2\right)}\left(\omega \right)}\right]}^{2}$$

Thus, if the ratio of the net active power flows $P_{1}^{\left(1\right)}\left(\omega \right)/P_{1}^{\left(2\right)}\left(\omega \right)$ increases, say 4 times, the ratio of the average reactive power flow $P_{2}^{\left(1\right)}\left(\omega \right)/P_{2}^{\left(2\right)}\left(\omega \right)$ grows only 2 times, etc. In other words, repartition of the net active power flow ($P_{1}^{\left(1\right)}\left(\omega \right),P_{1}^{\left(2\right)}\left(\omega \right)$) between two half-spaces of the waveguide is much more sensitive to changes in the penetration depths $\delta_{1}\left(\omega \right)/\lambda$ and $\delta_{2}\left(\omega \right)/\lambda$ than that of the average reactive power flow ($P_{2}^{\left(1\right)}\left(\omega \right),P_{2}^{\left(2\right)}\left(\omega \right)$) in the transverse direction $x_{2}$.

## 4. Correspondence between the SPP Electromagnetic Waves and the Proposed New SH Elastic Surface Waves

As it was stated before, the proposed new SH elastic surface waves can be considered an elastic analogue of the SPP electromagnetic surface waves propagating at a metal–dielectric interface. In fact, the mathematical models of both types of waves are formally identical. Therefore, it will be advantageous to identify explicitly the corresponding field variables in both domains, since the results obtained in one domain can be directly transferred to the other domain, alleviating thereby tedious from scratch derivations of the resulting analytical formulas (see Table 1).

As a result, the analytical formulas for all field variables analyzed in this paper, such as mechanical displacement $u_{3}\left(x_{2}\right)$, shear stresses $\tau_{23}\left(x_{2}\right),\tau_{13}\left(x_{2}\right)$, transverse wavenumbers $q_{1},q_{2}$, wavenumber k(ω), phase velocity $v_{p}\left(\omega \right)$, group velocity $v_{g}\left(\omega \right)$, penetration depths $\delta_{1}\left(\omega \right),{\;\delta }_{2}\left(\omega \right)$, net active power flows $P_{1}^{\left(1\right)}\left(\omega \right),P_{1}^{\left(2\right)}\left(\omega \right)$, average reactive power flows $P_{2}^{\left(1\right)}\left(\omega \right),P_{2}^{\left(2\right)}\left(\omega \right)$, as well as the dispersion relation can be readily transferred to the SPP domain by a simple substitution of the corresponding symbols.

In particular, Equations (10)–(37) developed in this paper in Section 3.1, Section 3.2, Section 3.3, Section 3.4, Section 3.5, Section 3.6, Section 3.7 and Section 3.8 are valid also (after simple replacement of the corresponding symbols) in the domain of SPP electromagnetic waves.

For example, phase velocity $v_{p}\left(\omega \right)$ of the SPP electromagnetic waves (see row 9 in Table 1) is expressed by the same formula as phase velocity $v_{p}\left(\omega \right)$ of the new SH elastic surface waves, providing that $s_{44}^{\left(1\right)}\left(\omega \right)$ and $s_{44}^{\left(2\right)}$ are substituted by $\varepsilon_{1}\left(\omega \right)$ and $\varepsilon_{2}$, respectively. The symbol $v_{2}$ corresponds to phase velocity of bulk SH waves in the conventional elastic material ($v_{2}=1/\sqrt{s_{44}^{\left(2\right)}\rho_{2}}$) and to bulk transverse electromagnetic waves in the dielectric ($v_{2}=1/\sqrt{\varepsilon_{2}\mu_{2}}$).

Interestingly, the crucial step in development of the quantitative model of the elastic metamaterial with a Drude-like elastic compliance (see Section 2.3) was the reverse transfer of an analytical equation developed in the electromagnetic domain (Equation B.2.5 in [20]) into the domain of the SH elastic waves (see Equation (7) in Section 2.3.3 and the accompanying discussion).

## 5. Numerical Results

### 5.1. Dispersion Curves

**Figure 9:**
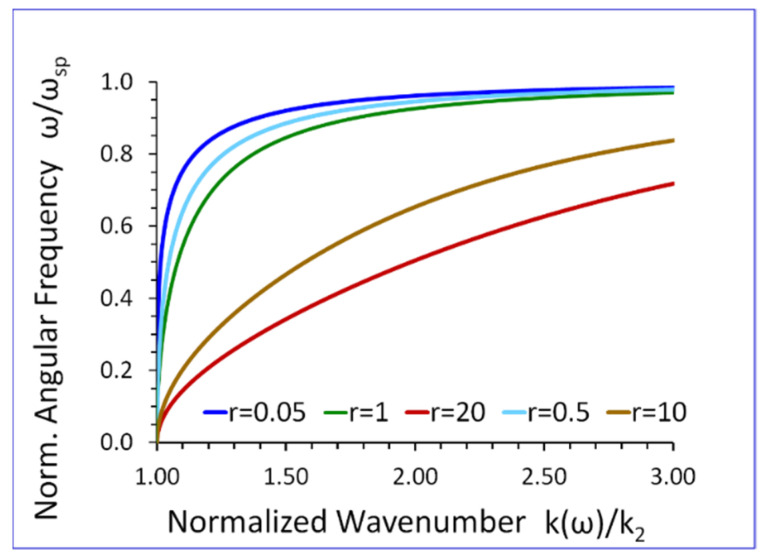
Normalized angular frequency $\omega /\omega_{sp}$ versus normalized wavenumber $k\left(\omega \right)/k_{2}$, for $r=\rho_{1}/\rho_{2}$ as a parameter ($s_{44}^{\left(2\right)}/s_{0}=1$).

Figure 9 presents the dispersion curves of the new surface acoustic wave. Using Equation (22), one can show that if ω→0, then k(ω)→0. On the other hand, when $\omega \to \omega_{sp}$ then the wavenumber k(ω)→∞, (see Figure 9).

### 5.2. Phase Velocity

**Figure 10:**
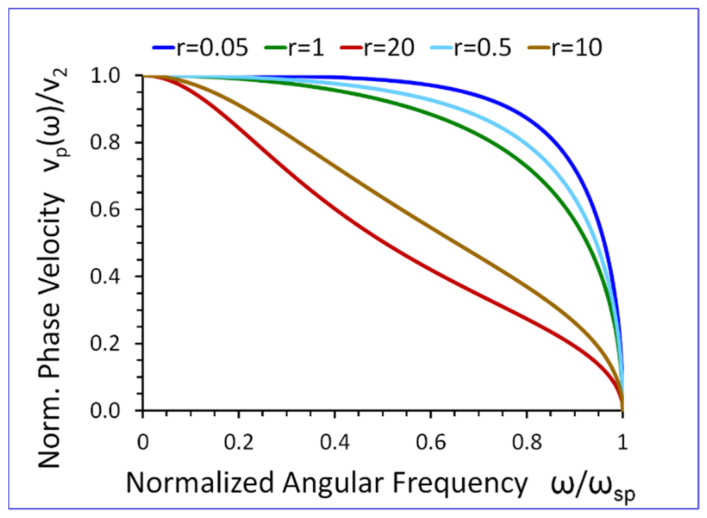
Normalized phase velocity $v_{p}\left(\omega \right)/v_{2}$ versus normalized angular frequency $\omega /\omega_{sp}$, for $r=\rho_{1}/\rho_{2}$ as a parameter ($s_{44}^{\left(2\right)}/s_{0}=1$).

Equation (25) shows that if ω→0, then $v_{p}\left(\omega \right)\to v_{2}$. On the other hand, when $\omega \to \omega_{sp}$, then the phase velocity $v_{p}\left(\omega \right)\to 0$, (see Figure 10).

### 5.3. Group Velocity

**Figure 11:**
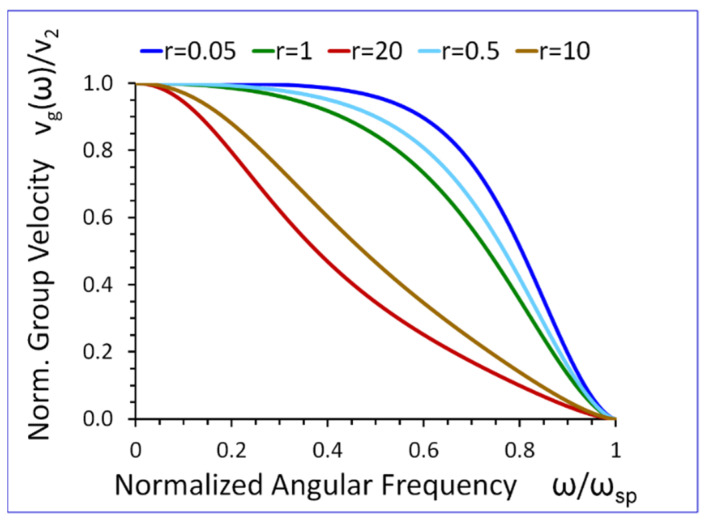
Normalized group velocity $v_{g}\left(\omega \right)/v_{2}$ versus normalized angular frequency $\omega /\omega_{sp}$, for $r=\rho_{1}/\rho_{2}$ as a parameter ($s_{44}^{\left(2\right)}/s_{0}=1$).

A closer look at Equation (26) reveals that if ω→0, then $v_{g}\left(\omega \right)\to v_{2}$ (see Figure 10. On the other hand, when $\omega \to \omega_{sp}$, then $v_{g}\left(\omega \right)\to 0$. Thus, phase $v_{p}\left(\omega \right)\;$and group $v_{g}\left(\omega \right)\;$velocities tend to the same limiting values for ω→0 and $\omega \to \omega_{sp}$, (see Figure 10 and Figure 11).

### 5.4. Penetration Depths in Both Half-Spaces

**Figure 12:**
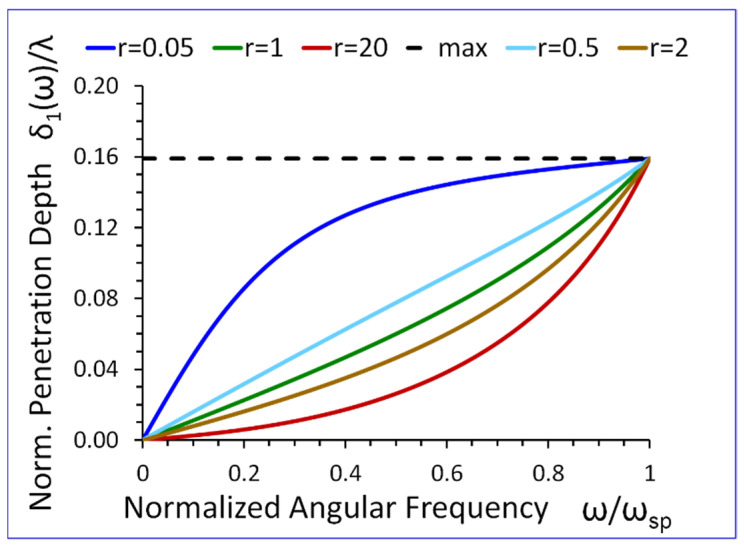
Normalized penetration depth $\delta_{1}\left(\omega \right)\;/\lambda$ in the metamaterial half-space, versus normalized angular frequency $\omega /\omega_{sp}$, for $r=\rho_{1}/\rho_{2}$ as a parameter ($s_{44}^{\left(2\right)}/s_{0}=1$).

Equation (27) shows that If the angular frequency ω→0 then the normalized penetration depth in the metamaterial half-space $\delta_{1}\left(\omega \right)/\lambda \to 0.\;$On the other hand, when $\omega \to \omega_{sp}$ then $\delta_{1}\left(\omega \right)/\lambda \to 1/2\pi .$ Thus, the normalized penetration depth $\delta_{1}\left(\omega \right)/\lambda$ in the metamaterial half-space is always subwavelength, i.e., $\delta_{1}\left(\omega \right)/\lambda <1/2\pi$, (see Figure 12).

**Figure 13:**
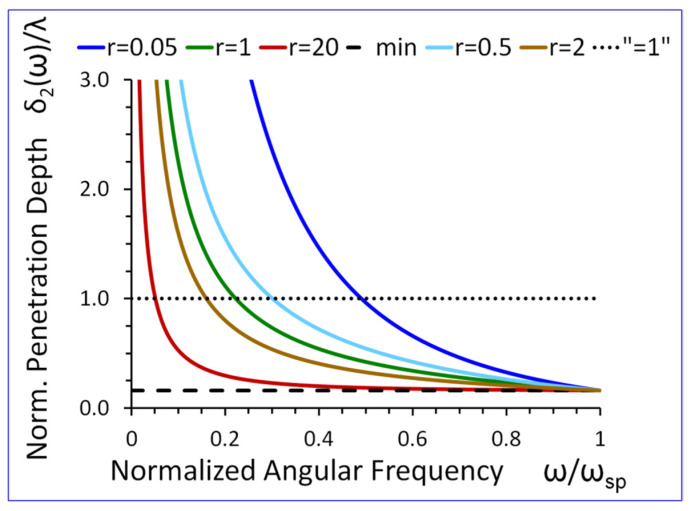
Normalized penetration depth $\delta_{2}\left(\omega \right)\;/\lambda$ in the conventional elastic half-space, versus normalized angular frequency $\omega /\omega_{sp}$, for $r=\rho_{1}/\rho_{2}$ as a parameter ($s_{44}^{\left(2\right)}/s_{0}=1$).

On the other hand (see Equation (28)), the normalized penetration depth in the conventional elastic half-space $\delta_{2}\left(\omega \right)/\lambda \to \infty$, if angular frequency ω→0. Similarly, when $\omega \to \omega_{sp}$ then $\delta_{2}\left(\omega \right)/\lambda \to 1/2\pi .$ As a result, the normalized penetration depth $\delta_{2}\left(\omega \right)/\lambda$ is higher than “1” (see dotted horizontal line in Figure 13) for low frequencies and subwavelength for high frequencies approaching the cut-off frequency $\omega_{sp}$, (see Figure 13).

### 5.5. Net Active Power Flow in the Direction of Propagation $x_{1}$

**Figure 14:**
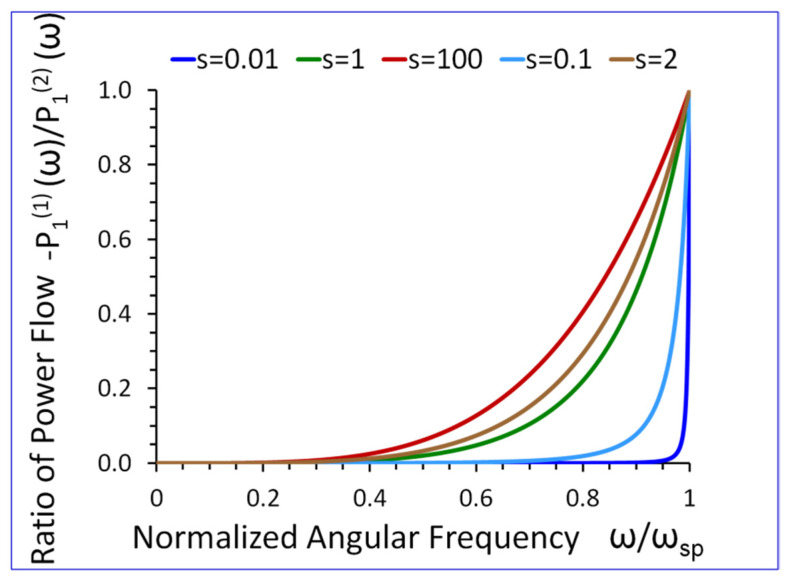
The ratio of net active power flows $-P_{1}^{\left(1\right)}\left(\omega \right)/P_{1}^{\left(2\right)}\left(\omega \right)$, in the direction of propagation $x_{1}$, versus normalized angular frequency $\omega /\omega_{sp}$, for $s=s_{44}^{\left(2\right)}/s_{0}$ as a parameter. $\rho_{1}$ and $\rho_{2}$ are arbitrary.

Using Equation (33) in conjunction with Equations (27) and (28), one can demonstrate that if ω→0 then $P_{1}^{\left(1\right)}\left(\omega \right)/P_{1}^{\left(2\right)}\left(\omega \right)\to 0$. On the other hand, if $\omega \to \omega_{sp}$, then $P_{1}^{\left(1\right)}\left(\omega \right)/P_{1}^{\left(2\right)}\left(\omega \right)\to -1$, (see Figure 14).

### 5.6. Average Reactive Power Flow in the Transverse Direction $x_{2}$

**Figure 15:**
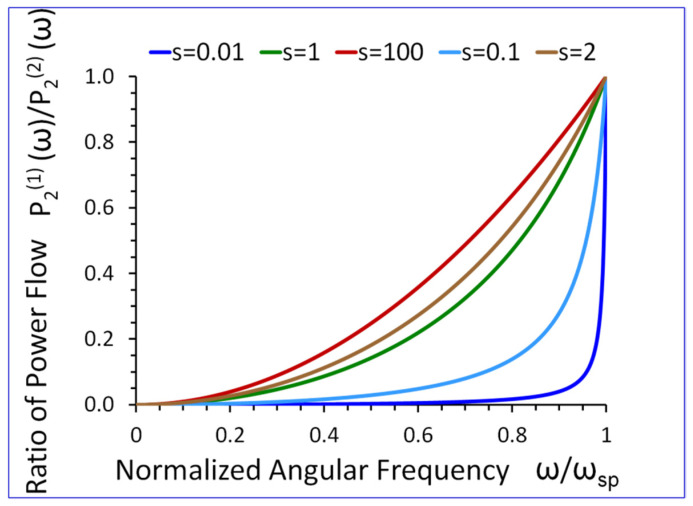
Ratio of average reactive power flows $P_{2}^{\left(1\right)}\left(\omega \right)/P_{2}^{\left(2\right)}\left(\omega \right)$, in the transverse direction $x_{2}$, versus normalized angular frequency $\omega /\omega_{sp}$, for $s=s_{44}^{\left(2\right)}/s_{0}$ as a parameter. $\rho_{1}$ and $\rho_{2}$ are arbitrary.

From Equation (36), we can conclude that if ω→0 then $P_{2}^{\left(1\right)}\left(\omega \right)$/$P_{2}^{\left(2\right)}\left(\omega \right)$ tends to zero. On the other hand, if $\omega \to \omega_{sp}$, then $P_{2}^{\left(1\right)}\left(\omega \right)/P_{2}^{\left(2\right)}\left(\omega \right)\to 1$, (see Figure 15).

## 6. Discussion

Elastic surface waves propagating in metamaterial waveguides were subject of a number of papers that analyzed the Rayleigh surface waves at the solid-vacuum interface [24], Scholte interfacial waves at the solid-liquid interface [25], shear horizontal waves on a semi-infinite half-space loaded with a metasurface [26,27] or Love surface waves in waveguides loaded with a resonant metasurface [28].

The possibility of the existence of elastic SH waves propagating at the interface of two elastic half-spaces, one of which is an elastic metamaterial, was briefly announced in one of the author’s previous works [29]. However, the present paper differs significantly from the former paper presented in [29]. In particular, in the present study:

(1) A general theory of the new SH elastic surface waves propagating at an elastic interface has been developed from first physical principles;
(2) All considered field variables are normalized, e.g., we use the normalized angular frequency $\omega /\omega_{sp}$, normalized wavenumber $k\left(\omega \right)/k_{2}$ etc.;
(3) The influence of the density of both half-spaces on the characteristics of the new elastic SH wave is taken into consideration;
(4) New analytical formulas for the penetration depths $\delta_{1}\left(\omega \right)/\lambda$ and $\delta_{2}\left(\omega \right)/\lambda$ were established. The newly developed formulas can be of significant practical importance in design of devices in the domain of SPP and in the domain of new SH elastic surface waves;
(5) A new quantitative model of the elastic metamaterial with a Drude-like elastic compliance $s_{44}^{\left(1\right)}\left(\omega \right)$ has been developed.

It should be emphasized that all the five developments mentioned above have been included in the present paper and were not yet published elsewhere.

Our former research [30] on elastic surface waves propagating in conventional elastic waveguides showed that SH surface waves, such as Love surface waves [31], share many common properties with waves in other domains of physics, such as TM (Transverse Magnetic) modes in optical planar waveguides or wave function of quantum particles in a potential well. However, the present paper was mostly influenced by recent developments in the domain of elastic metamaterials and SPP electromagnetic surface waves propagating at the metal-dielectric interface [32].

In this paper, we demonstrated that the ultrasonic analogue of SPP electromagnetic waves can exist in elastic waveguides consisting of two elastic half-spaces, providing that one of the elastic half-spaces is an elastic metamaterial with a negative elastic compliance $s_{44}^{\left(1\right)}\left(\omega \right)$ that corresponds to the dielectric function ε(ω) in Drude’s model of metals. These two types of waves are described by formally identical mathematical models and, therefore, have similar (1) distribution field variables and (2) dispersion equation.

The dispersion curves of the new SH elastic surface wave, shown in Figure 9, have the characteristic property that the wavenumber k(ω) tends to infinity k(ω)→∞, when the wave angular frequency ω approaches the cuff-of frequency $\omega_{sp}$. Since λ=2π/k, the wavelength λ of the new SH elastic surface wave tends to zero λ→0 when $\omega \to \omega_{sp}$. This phenomenon can be exploited in the subwavelength near field ultrasonic imaging.

Another very intriguing property of the new SH elastic surface waves is that their phase $v_{p}\left(\omega \right)$ and group $v_{g}\left(\omega \right)$ velocities tend to zero when the wave frequency approaches the cut-off frequency $\omega \to \omega_{sp}$ (see Figure 10 and Figure 11). This property is of key importance in the potential applications of the new SH elastic surface wave in ultrasonic sensors with extremely large mass sensitivity, which can give rise to a new generation of biosensors and chemosensors with unprecedented sensitivity.

This paper contains several new original formulas which to the best of our knowledge were not yet published in the literature, namely:

- Relation for the product of penetration depths $\delta_{1}\left(\omega \right)$, $\delta_{2}\left(\omega \right)$ in two half-spaces of the waveguide (Equation (30));
- Relation between net active power flows $P_{1}^{\left(1\right)}\left(\omega \right)$, $P_{1}^{\left(2\right)}\left(\omega \right)$ in the direction of propagation $x_{1}$ and penetration depths $\delta_{1}\left(\omega \right)$, $\delta_{2}\left(\omega \right)$ in two half-spaces of the waveguide (Equation (33));
- Relation between average reactive power flows $P_{2}^{\left(1\right)}\left(\omega \right)$, $P_{2}^{\left(2\right)}\left(\omega \right)$ in the transverse direction $x_{2}$ and penetration depths $\delta_{1}\left(\omega \right)$, $\delta_{2}\left(\omega \right)$ in two half-spaces of the waveguide (Equation (36));
- Relation between net active power flows $P_{1}^{\left(1\right)}\left(\omega \right)$, $P_{1}^{\left(2\right)}\left(\omega \right)$ in the direction of propagation $x_{1}$ and average reactive power flows $P_{2}^{\left(1\right)}\left(\omega \right)$, $P_{2}^{\left(2\right)}\left(\omega \right)$ in the transverse direction $x_{2}$ of the waveguide (Equation (37)).

All new equations mentioned above, which were developed in the elastic domain, can be directly transferred into the domain of SPP electromagnetic surface waves, using to this end Table 1 presented in Section 4. In particular, the relation between the penetration depths $\delta_{1}\left(\omega \right)$, $\delta_{2}\left(\omega \right)$ in two half-spaces of the waveguide (Equation (30)) can be useful for designers of SPP electromagnetic sensors, in selection of proper wave frequency providing high subwavelength concentration of energy in the dielectric material of the waveguide leading to long range propagation of SPP waves.

Similarly, the new relations between the power flows and the penetration depths in two half-spaces of the waveguide (Equations (33) and (36)) indicate that the proper control of the net active power flow in the direction of propagation may be very important in achieving high sensitivity of long range SPP sensors with low losses.

The results presented in Figure 9, Figure 10, Figure 11, Figure 12, Figure 13, Figure 14 and Figure 15 reveal that the densities $\rho_{1},\;\rho_{2}$, in both half-spaces of the waveguide, have a profound impact on all parameters of the proposed new SH elastic surface waves. For example, if $\rho_{1}/\rho_{2}=1$, the penetration depth in the metamaterial half-space $\delta_{1}\left(\omega \right)$ is ~43 times smaller than the wavelength λ of the wave, at $\omega /\omega_{sp}=0.2$ (see green curve Figure 12). By contrast, if $\rho_{1}/\rho_{2}=20$ the penetration depth $\delta_{1}\left(\omega \right)$ decreases significantly and is ~167 times smaller than the wavelength λ (see red curve in Figure 12).

Therefore, since the densities $\rho_{1},\;\rho_{2}$ correspond to magnetic permeabilities $\mu_{1},\;\mu_{2}$ in SPP electromagnetic waveguides (see rows 6 and 7 in Table 1 in Section 4) it implies that we can also effectively shape the characteristics of SPP electromagnetic waves by analogous adjustment of $\mu_{1}$ and$\;\mu_{2}$.

On the other hand, due to strong formal similarities between the new SH elastic surface waves and SPP electromagnetic surface waves it may be possible in future to transfer many fascinating newly discovered SPP phenomena, such as cloaking [14], trapping (zero group velocity) [13] and topological protection [15] into the domain of elastic metamaterials using to this end the new SH elastic surface waves, proposed in this paper.

As a result, the proposed new SH elastic surface waves can open new possibilities to control wave phenomena in elastic solids and can constitute the basis for a new generation of modern devices in the domain of sensors, acoustic imaging, and signal processing.

Using recently discovered elastic hyperbolic metamaterials [33] we can achieve subwavelength imaging by amplification of the evanescent waves scattered from the object, which contain information about fine details of the object. The evanescent waves are not only amplified but also are converted to propagation modes, which can be focused in a far zone of the hyperbolic superlens. However, the same amplification of the evanescent waves and subwavelength imaging can be achieved with the proposed new SH elastic surface waves, but in a simpler way. In fact, the elastic hyperbolic metamaterials are quite complicated since they require that the mass density of the hyperbolic metamaterial must be simultaneously anisotropic and negative [34]. By contrast, using the new SH elastic surface waves we can also achieve subwavelength imaging and amplification of the evanescent waves but in a much simpler way. In fact, two half-spaces of the waveguide supporting the new SH waves are always isotropic and only one metamaterial half-space must exhibit a negative Drude-like elastic compliance.

Finally, we must address the issue of losses that will inevitably occur in waveguides of the proposed new SH acoustic surface waves. Interestingly, the problem of losses was solved in SPP devices by the introduction of a multilayer waveguide structure. For example, a very thin layer (25 nm) of lossy metal (Au) was sandwiched between two low loss dielectrics (SU-8 polymer) provided a 5 mm long sensor [35]. The presence of losses may also affect efficiency of specific wave phenomena occurring in metamaterial waveguides, such as zero group velocity. In fact, in reference [36] it was shown that the minimal group velocity that can be achieved in waveguides with losses is always higher than zero.

Moreover, the presence of losses can limit the maximum value of the wavenumber k(ω) of the SH surface wave propagating at the boundary of the elastic half-space and the metamaterial half-space with Drude-like elastic compliance. This may limit the resolution of Drude-type metamaterial superlenses used in near-field acoustic imaging.

This paper is a clear example of the multidisciplinary research that can bring new valuable and sometimes unexpected physical insight on the physical phenomena occurring in two domains of physics, i.e., theory of elasticity and electromagnetism.

It will be advantageous in future research to extend the analysis of the new SH elastic surface waves on waveguides with losses as well as to design a model of a biosensor based on the analogy with SPP electromagnetic devices [35,37].

## 7. Conclusions

Based on the results of research presented in this paper, we can draw the following detailed conclusions:

1. The new SH elastic surface waves can be considered as an elastic analogue of the electromagnetic SPP waves, due to strong formal similarities of their mathematical models (Table 1 in Section 4);
2. The new SH elastic surface waves can exist at the interface of two elastic half-spaces one of which is an elastic metamaterial with a negative compliance $s_{44}^{\left(1\right)}\left(\omega \right)\cdot s_{44}^{\left(2\right)}<0$ (Equation (21));
3. The phase velocity $v_{p}\left(\omega \right)$ of the new SH ultrasonic surface waves is antiparallel to the net active power flow $P_{1}^{\left(1\right)}\left(\omega \right)$ in the metamaterial half-space and parallel to the net active power flow $P_{1}^{\left(2\right)}\left(\omega \right)$ in the conventional elastic half-space;
4. The net active power flows $P_{1}^{\left(1\right)}\left(\omega \right),\;P_{1}^{\left(2\right)}\left(\omega \right)$ of the new SH elastic surface waves, in both half-spaces, are antiparallel along the direction of propagation $x_{1}$, (Equations (31) and (32));
5. An average reactive power flows $P_{2}^{\left(1\right)}\left(\omega \right),\;P_{2}^{\left(2\right)}\left(\omega \right)$, in the transverse direction $x_{2}$, have the same sign (+) corresponding to the inductive type of the reactive power, oscillating between two half-spaces of the waveguide (Equations (34) and (35));
6. The penetration depth $\delta_{1}\left(\omega \right)$ of the new SH elastic surface waves in the metamaterial half-space is always smaller than that in the conventional elastic half-space $\delta_{2}\left(\omega \right)$, i.e., $\delta_{1}\left(\omega \right)<\delta_{2}\left(\omega \right)$ (Figure 12 and Figure 13);
7. The ratio of the net active power flows $P_{1}^{\left(1\right)}\left(\omega \right)/P_{1}^{\left(2\right)}\left(\omega \right)$ and the corresponding ratio of the average reactive power flows $P_{2}^{\left(1\right)}\left(\omega \right)/P_{2}^{\left(2\right)}\left(\omega \right)$ are intimately related to the ratio of the penetration depths $\delta_{1}\left(\omega \right)/\delta_{2}\left(\omega \right)$ in both half-spaces of the waveguide (Equations (33) and (36) and Figure 14 and Figure 15);
8. The ratio of the net active power flows $P_{1}^{\left(1\right)}\left(\omega \right)/P_{1}^{\left(2\right)}\left(\omega \right)$ and the corresponding ratio of the average reactive power flows $P_{2}^{\left(1\right)}\left(\omega \right)/P_{2}^{\left(2\right)}\left(\omega \right)$ are not independent since they are related via Equation (37);
9. The penetration depth (see Figure 12 and Figure 13) in both elastic half-spaces of the waveguide is deeply subwavelength. Therefore, the new SH elastic surface waves can find applications in sensors of extremely high mass sensitivity, superlensing, and in near field acoustic microscopy with a subwavelength resolution and imaging. These are very exciting applications of the newly discovered SH ultrasonic waves;
10. Several new formulas (Equations (30), (33), (36), and (37)) developed in this paper may also be useful in the design of long range SPP waveguides with low propagation losses;
11. The densities $\rho_{1},\;\rho_{2}$, in both half-spaces of the waveguide, have a profound impact on all parameters of the proposed new elastic surface waves (Figure 9, Figure 10, Figure 11, Figure 12, Figure 13, Figure 14 and Figure 15). Therefore, by virtue of Table 1 in Section 4, we can also effectively shape the characteristics of SPP electromagnetic waves by analogous adjustment of the corresponding magnetic permeabilities $\mu_{1}$ and$\;\mu_{2}$;
12. Newly discovered SPP phenomena, such as cloaking, trapping (zero group velocity), and topological protection can be transferred into the domain of elastic metamaterials using to this end the new SH elastic surface waves, proposed in this paper. This may open new fascinating possibilities to control wave phenomena in the domain of elastodynamics.

It should be emphasized that due to their close similarity with the electromagnetic SPP waves the proposed new SH elastic surface waves are characterized by a large confinement of acoustic energy near the surface. For this reason, the proposed new SH elastic surface waves can constitute a basis of a new generation of ultrasonic sensors with a giant mass sensitivity. For example, the new SH elastic surface waves can find applications in:

- ultrasonic sensors with extremely high mass sensitivity;
- biosensors and chemosensors;
- sub-wavelength ultrasonic microscopy and imaging.

Because of its interdisciplinary character, the present paper can be of interest for a broad spectrum of researchers and engineers working in different domains of science and technology, such as acoustics, optics, elastic metamaterials, ultrasonic sensors, biosensors, and chemosensors.

## Figures and Tables

**Table 1:** Correspondence between field variables of the SPP electromagnetic waves propagating in metal–dielectric waveguides and the proposed new SH elastic surface waves propagating in metamaterial waveguides.

No	SPP Electromagnetic Surface Waves in Metal–Dielectric Waveguides		New SH Elastic Surface Waves in Metamaterial Waveguides	
	Property	Implementation	Implementation	Property
1	Longitudinal electric field	$E_{1}$	$\tau_{23}$	Shear horizontal SH mechanical stress
2	Transverse electric field	$E_{2}$	$\tau_{13}$	Shear mechanical stress
3	transverse magnetic field	$H_{3}$	$v_{3}=-j\omega u_{3}$	SH particle velocity $v_{3}=\partial u_{3}/\partial t$
4	Dielectric function in metal	$\varepsilon_{1}\left(\omega \right)$	$s_{44}^{\left(1\right)}\left(\omega \right)$	Elastic compliance in metamaterial half-space
5	Dielectric function in dielectric	$\varepsilon_{2}$	$s_{44}^{\left(2\right)}$	Elastic compliance in conventional half-space
6	Magnetic permeability in metal	$\mu_{1}$	$\rho_{1}$	Density of metamaterial half-space
7	Magnetic permeability in dielectric	$\mu_{2}$	$\rho_{2}$	Density of conventional half-space
8	Wavenumber for $\mu_{1}/\mu_{2}=1$	$k\left(\omega \right)=k_{2}\sqrt{\frac{\varepsilon_{1}\left(\omega \right)}{\varepsilon_{1}\left(\omega \right)+\varepsilon_{2}}}$	$k\left(\omega \right)=k_{2}\sqrt{\frac{s_{44}^{\left(1\right)}\left(\omega \right)}{s_{44}^{\left(1\right)}\left(\omega \right)+s_{44}^{\left(2\right)}}}$	Wavenumber for $\rho_{1}/\rho_{2}=1$
9	Phase velocity of SPP electromagnetic waves	$v_{p}\left(\omega \right)=v_{2}\sqrt{\frac{\varepsilon_{1}\left(\omega \right)+\varepsilon_{2}}{\varepsilon_{1}\left(\omega \right)}}$	$v_{p}\left(\omega \right)=v_{2}\sqrt{\frac{s_{44}^{\left(1\right)}\left(\omega \right)+s_{44}^{\left(2\right)}}{s_{44}^{\left(1\right)}\left(\omega \right)}}$	Phase velocity of new SH elastic surface waves
10	Complex Poynting vector in propagation direction $x_{1}$	$P_{1}=\frac{1}{2}E_{2}\times H_{3}^{*}$	$P_{1}=-\frac{1}{2}\tau_{13}v_{3}^{*}$	Complex Poynting vector in propagation direction $x_{1}$
11	Complex Poynting vector in transverse direction $x_{2}$	$P_{2}=\frac{1}{2}E_{1}\times H_{3}^{*}$	$P_{2}=-\frac{1}{2}\tau_{23}v_{3}^{*}$	Complex Poynting vector in transverse direction $x_{2}$
12	Wave impedance $Z_{TM}=E_{2}/H_{3}$, TM modes	$Z_{TM}^{-1}=v_{p}\left(\omega \right)\{\begin{array}{c} \varepsilon_{1}\left(\omega \right),\;metal\\ \varepsilon_{2},dielec \end{array}$	$Z_{s}^{-1}=v_{p}\left(\omega \right)\{\begin{array}{c} s_{44}^{\left(1\right)}\left(\omega \right),meta.\;\\ s_{44}^{\left(2\right)},conven. \end{array}$	Wave impedance $Z_{s}=-\tau_{13}/v_{3}$, elastic surface waves

## Data Availability

Data are contained within the article.
